# Schedule-Dependent Antiangiogenic and Cytotoxic Effects of Chemotherapy on Vascular Endothelial and Retinoblastoma Cells

**DOI:** 10.1371/journal.pone.0160094

**Published:** 2016-07-28

**Authors:** Ursula Winter, Hebe A. Mena, Soledad Negrotto, Eloisa Arana, Guillem Pascual-Pasto, Viviana Laurent, Mariona Suñol, Guillermo L. Chantada, Angel M. Carcaboso, Paula Schaiquevich

**Affiliations:** 1 Clinical Pharmacokinetics Unit, Hospital de Pediatría JP Garrahan, Buenos Aires, Argentina; 2 National Scientific and Technical Research Council, CONICET, Buenos Aires, Argentina; 3 Experimental Thrombosis Laboratory, IMEX, National Academy of Medicine, Buenos Aires, Argentina; 4 Inmunogenetics Laboratory, INIGEM, University of Buenos Aires, Buenos Aires, Argentina; 5 Developmental tumor biology Laboratory and Department of Pediatric Hematology and Oncology, Hospital Sant Joan de Deu, Barcelona, Spain; 6 Pathology, Hospital Sant Joan de Deu, Barcelona, Spain; 7 Hospital de Pediatría JP Garrahan, Buenos Aires, Argentina; European Institute of Oncology, ITALY

## Abstract

Current treatment of retinoblastoma involves using the maximum dose of chemotherapy that induces tumor control and is tolerated by patients. The impact of dose and schedule on the cytotoxicity of chemotherapy has not been studied. Our aim was to gain insight into the cytotoxic and antiangiogenic effect of the treatment scheme of chemotherapy used in retinoblastoma by means of different *in vitro* models and to evaluate potential effects on multi-drug resistance proteins. Two commercial and two patient-derived retinoblastoma cell types and two human vascular endothelial cell types were exposed to increasing concentrations of melphalan or topotecan in a conventional (single exposure) or metronomic (7-day continuous exposure) treatment scheme. The concentration of chemotherapy causing a 50% decrease in cell proliferation (IC50) was determined by MTT and induction of apoptosis was evaluated by flow cytometry. Expression of *ABCB1*, *ABCG2* and *ABCC1* after conventional or metronomic treatments was assessed by RT-qPCR. We also evaluated the *in vivo* response to conventional (0.6 mg/kg once a week for 2 weeks) and metronomic (5 days a week for 2 weeks) topotecan in a retinoblastoma xenograft model. Melphalan and topotecan were cytotoxic to both retinoblastoma and endothelial cells after conventional and metronomic treatments. A significant decrease in the IC50 (median, 13-fold; range: 3–23) was observed following metronomic chemotherapy treatment in retinoblastoma and endothelial cell types compared to conventional treatment (p<0.05). Metronomic topotecan or melphalan significantly inhibited *in vitro* tube formation in HUVEC and EPC compared to vehicle-treated cells (p<0.05). Both treatment schemes induced apoptosis and/or necrosis in all cell models. No significant difference was observed in the expression of *ABCB1*, *ABCC1* or *ABCG2* when comparing cells treated with melphalan or topotecan between treatment schedules at the IC50 or with control cells (p>0.05). In mice, continuous topotecan lead to significantly lower tumor volumes compared to conventional treatment after 14 days of treatment (p<0.05). Continuous exposure to melphalan or topotecan increased the chemosensitivity of retinoblastoma and endothelial cells to both chemotherapy agents with lower IC50 values compared to short-term treatment. These findings were validated in an *in vivo* model. None of the dosing modalities induced multidrug resistance mechanisms while apoptosis was the mechanism of cell death after both treatment schedules. Metronomic chemotherapy may be a valid option for retinoblastoma treatment allowing reductions of the daily dose.

## Introduction

Patients with intraocular retinoblastoma, the most common eye cancer of childhood, are usually offered conservative therapy with chemotherapy in order to avoid enucleation [1,2]. The standard conservative therapy involves intravenous or local (ophthalmic artery chemosurgery or intravitreal injections) chemotherapy including melphalan, carboplatin and topotecan based on the extensive knowledge of the antitumor activity of these agents[3–6]. Despite the promising effect of new local treatments on ocular survival, these treatments are not devoid of retinal toxicity causing vision loss and relapses mainly occurring in the vitreous, known as vitreous seeds[3,7,8]. For those eyes that relapse after intravitreal chemotherapy, it is not possible to further increase the chemotherapy dose because of unacceptable retinal toxicity that would impair vision, and thus no alternative treatments are available[9–12]. Therefore, new agents or alternative schedules of active drugs are needed for tumor control. Chemotherapy treatment schedules, specifically the dose and frequency of administration, have historically been established empirically based on the ophthalmologist’s observations of tumor response for each individual patient during follow-up. Usually, the schedules consist of intravitreal injection or infusion through the ophthalmic artery at the maximum tolerated dose (MTD)[3,4,11]. Patients with extraocular dissemination of retinoblastoma carry a dismal prognosis. High dose chemotherapy with stem cell rescue may cure a proportion of these children but when CNS dissemination occurs[13], survival is uncommon. Innovative treatment alternatives are needed since it is not possible to further increase the dose of these intensive regimens.

Metronomic chemotherapy is a treatment modality of continuous and repetitive administration of chemotherapy at relatively lower doses compared to maximum tolerated schedules[14–16]. Although information is limited, a potential advantage is related to avoiding severe systemic adverse events because of the lower blood concentrations after each dose while obtaining the same clinical outcome as after the maximum tolerated dose. Accumulating evidence suggests that metronomic chemotherapy is a promising treatment schedule for certain pediatric solid tumors in terms of efficacy and safety based on clinical and preclinical studies[17–21]. Interestingly, the development of metronomic treatment has mainly been related to targeting tumor-associated vascular development as part of tumor control[14,15,22,23]. Although the mechanism has not been elucidated, the activity of metronomic schemes is not only associated with the antiangiogenic activity, but also, with a direct antitumor effect [14,15]. Nonetheless, data on cytotoxic activity of routinely used agents for retinoblastoma treatment in endothelial vascular and tumor cells are scarce.

There is a clear limitation to conducting metronomic studies in intraocular retinoblastoma due to the fact that vitreous seeds can not be repetitively punctured by intravitreal injections for continuous or daily treatment because of risk of extraocular seedings[24]. Nevertheless, this problem could be solved by means of a sustained release device loaded with chemotherapy and implanted in the vitreous as the local injection is the preferred route for targeting the ocular in cases of intraocular retinoblastoma[1,25]. Continuous chemotherapy might be of benefit mainly for intraocular retinoblastoma patients with vitreous seeds recently classified as clouds (massive tumor cells in the vitreous) as they need more intravitreal injections and cumulative dose of chemotherapy for tumor control compared to other types of seeds[8]. In cases of disseminated disease, oral chemotherapy would be an option, although close monitoring should be performed[19,20,26]. One of the main concerns about continuous exposure to antineoplastic agents is the association with overexpression of adenosine-triphosphate binding cassette (ABC) transporters as one of the many mechanisms likely to contribute to multiple drug resistance[27–29]. This superfamily of proteins including P-glycoprotein, breast cancer resistance protein and multiple drug resistance protein 1 (gene symbols *ABCB1*, *ABCG2*, and *ABCC1*, respectively) extrudes a range of structurally unrelated compounds impairing intracellular drug accumulation thereby affecting chemotherapy efficacy with potential implications for drug resistance[29]. So far, no previous studies have been published on the influence of schedule exposure (continuous metronomic versus single-dose) to chemotherapy on the expression of these efflux transporter proteins in retinoblastoma cells.

Therefore, with the aim of finding new treatment strategies in retinoblastoma, we assessed the effect of melphalan and topotecan schedule of treatment on the viability and angiogenic activity of late outgrowth endothelial progenitor cells (EPC) or mature endothelial cells from macrovascular endothelium (HUVEC). We also evaluated whether single-dose and continuous exposure to these chemotherapy agents affected the expression of ABC transporters in retinoblastoma and endothelial cells for potential implications regarding drug resistance mechanisms.

## Materials and Methods

### Ethics statement

The present study was approved by Coordinación de Investigación Hospital de Pediatría JP Garrahan, Buenos Aires, Argentina (protocol number 790). Patient-derived retinoblastoma cell models were obtained under an Institutional Review Board-approved protocol and written informed consent (M-1608-C) at Hospital Sant Joan de Deu Barcelona, Spain. The Institutional Review Board of the National Academy of Medicine, Argentina approved the protocol for obtaining EPC and HUVEC and written informed consent was obtained from the mothers before sample collection.

### Chemicals and reagents

Melphalan and topotecan were kindly provided by Eriochem and Asofarma, respectively. Stock solutions of melphalan (2mg/ml) and topotecan (1mg/ml) were prepared in methanol and stored at -20C. Serial dilutions were performed with culture medium before use.

Proliferation of cells was determined by 3-(4, 5-dimethylthiazol-2-yl)-2, 5-diphenyl tetrazolium bromide (MTT) colorimetric assay purchased from Sigma-Aldrich, St. Louis, MO, USA. Matrigel was obtained from BD bioscience.

### Cell types and culture

Commercial retinoblastoma cell lines Y79 and WERI-RB1 were obtained from the American Type Culture Collection (ATCC HTB-18 and HTB-169, respectively, Manassas, VA). Cells were cultured in RPMI-1640 culture media (Invitrogen Life Technologies, Carlsbad, CA, USA) supplemented with fetal bovine serum (FBS, Greiner Bio-one, Wemmel, Belgium), 2 mM L-glutamine, 1.5 gr/l NaHCO3, 4.5gr/l glucose, 10 mM HEPES and 1 mM sodium pyruvate.

Human umbilical vein endothelial cells (HUVEC) and late outgrowth endothelial progenitor cells (EPC) were obtained from human umbilical cord veins and blood respectively, from healthy women who had full-term deliveries as previously described[30]. In addition, endothelial cells were maintained in endothelial growth medium-2 (EGM2) from Lonza (Walkersville, MD).

Finally, patient-derived retinoblastoma cell models HSJD-RBT-7 and HSJD-RBT-8 were obtained from enucleated eyes of two female patients with no germline RB1 mutations at Hospital Sant Joan de Deu Barcelona, Spain. To obtain the tumor samples from the enucleated eyes, a window was opened in the sclera with a blade and the retinal tumor was retrieved with dissection tweezers. The HSJD-RB-007 model was established from a treatment-naive tumor (upfront enucleation), whereas HSJD-RB-008 had received extensive chemotherapy before enucleation consisting of five tandem doses of topotecan and melphalan delivered by ophthalmic artery chemosurgery. Both cell models result from unilateral patients with intraocular disease, no metastasis, and without treatment of external beam radiotherapy. Tumors were manually disaggregated and cultured as tumorspheres, in serum-free conditions as previously described for the culture of pediatric glioma stem cells[31]. All cultures were maintained at 37°C in a humidified 95% air and 5% CO2 atmosphere.

### Conventional and metronomic chemotherapy-induced cytotoxicity

First, cells were counted with a hemocytometer, seeded in 96-well plates, and cultured for 24 h.

To assess the effect of conventional treatment of chemotherapy (i.e, to mimic the clinical scenario of single exposure at the maximum tolerated dose, MTD), each of the four retinoblastoma cell models and two endothelial cell types (HUVEC, EPC) were exposed to different concentrations of topotecan (0.001–10.000nM) or melphalan (0.001–1000μM). MTT assay was performed 72 h later.

In the metronomic schedule (low tolerated dose, LTD), cells were exposed to topotecan (0.0001 to 1000nM) or melphalan (0.0001 to 100μM) daily for a total of 7 days. To maintain a constant concentration for the whole week, every 24 hours the medium was replaced by fresh medium with the same concentration of chemotherapy in each well. MTT assays were performed at the end of day 7.

In all cases, wells with medium and PBS served as controls and five independent assays were performed in triplicate for each concentration of melphalan or topotecan. Thereafter, the number of viable cells was assessed using the MTT assay as previously reported elsewhere. Then, the concentration of melphalan or topotecan that caused a 50% decrease in cell proliferation (MTD-IC50 and LTD-IC50) was calculated using GraphPad Prism v.5 (GraphPad Software Inc, La Jolla, CA).

### Apoptosis assay

To study the effect of the schedule of melphalan and topotecan on apoptosis, retinoblastoma and endothelial cells were stained with annexin V and propidium iodide (PI) using the Annexin V- FITC Apoptosis Detection Kit II (BD Pharmingen, San Diego, CA). Staining was performed as previously described elsewhere. Briefly, retinoblastoma and endothelial cells were seeded and treated with each chemotherapy agent at the corresponding IC50 in a conventional or metronomic fashion. After washing, cells were incubated with FITC-annexin V for 15min in the dark and resuspended in PI solution for immediate flow cytometry analysis using a FACSAria (BD Biosciences). In addition, nuclear morphology was analyzed with fluorescence microscopy after acridine orange (100μg/ml) and propidium iodide (100μg/ml, Sigma Aldrich) double-staining.

### In vitro Matrigel assay

After exposing cells to conventional and metronomic treatments, HUVEC and EPC cells were detached with trypsin–EDTA and seeded at a density of 1.5 x 10^4^ viable cells (trypan blue exclusion) per well on 96-well plates coated with growth factor-reduced basement membrane matrix (Geltrex^™^; Invitrogen) in triplicate wells for each condition. After 18 hours of incubation at 37°C, tube formation was examined by phase-contrast microscopy (Leica microscope) and the total number of branch points and tube length was quantified on the entire well surface by using ImageJ software.

### Real-time Quantitative Reverse Transcription PCR

Real-time quantitative polymerase chain reaction (qPCR) was used to quantitate the mRNA levels of *ABCB1*, *ABCC1*, and *ABCG2* in Y79 and EPC as an exploratory effect of continuous treatment with chemotherapy in retinoblastoma and endothelial cell types. In the conventional treatment schedule, cells were exposed to drug-free medium or to melphalan or topotecan at the MTD-IC50 for 72 h. In the continuous treatment group, cells were treated with drug-free medium (control cells) or with chemotherapy at the LTD-IC50 for 7 days. After ending treatments, total RNAs from all cell types were isolated using PureLink RNA Mini Kit (Invitrogen) according to the manufacturer's instructions. RNA pellets were resuspended in a 30-μl final volume of DEPC-treated water and stored at -80°C until use. RNA was quantified using a nanodrop spectrophotometer and after quantification was reverse transcribed to obtain cDNA with SuperScript III First-Strand (Invitrogen) according to the standard protocol.

The transcription levels of ABC transporters and GAPDH (housekeeping gene) was performed by quantitative RT-PCR using TaqMan gene expression assays (Applied Biosystems). Primers for real-time PCR were designed using Primer Express software version 2.0 (Applied Biosystems) and synthesized by Invitrogen. The sequences of the primers were the following:

***ABCB1***-*forward*: 5′- CGCTATGGCCGAGAAGATGT-3′ and *ABCB1-reverse*: 5′- CCATGGATGAGATTGAAA-3′; *probe* 5′- CCAGGGTGTCAAATTTATGAGG -3′; ***ABCC1***-*forward*: 5′- ATGGCGATGAAGACCAAGAC-3′ and *ABCC1*-*reverse*: 5′-AGCACCTTGTCCTTGAATGC-3′, *probe TGGCCCACATGAAGAGCAAAGACA* ***ABCG2***-*forward*: 5′- ATCTTGGCTGTCATGGCTTC-3′ and *ABCG2*-*reverse*: 5′- TCCTGTTGCATTGAGTCCTG -3′ *probe* 5′- CGATATGGATTTACGGCTTTGCAGC -3′.

The threshold cycle (Ct) of each target product was determined and normalized against that of the housekeeping gene GAPDH. All reactions were performed in triplicate using 20μL samples containing 250ng cDNA. Thermocycling conditions were heating for 2 min at 50°C and 10 min at 95°C, followed by 40 cycles of amplification (15 s at 95°C and 1 min at 60°C).

### Retinoblastoma xenografts in mice and drug-treatment

Female athymic nude mice, weighing 18–22 g were provided by Envigo (Barcelona, Spain). Animals were housed in pathogen-free cages in a light and temperature-controlled room and were allowed to unrestricted autoclaved food and water access. Animal studies were performed according to the Institutional and European guidelines (EU Directive 2010/63/EU) and complied with the tenets of the Association for Research in Vision and Ophthalmology for the use of animals in ophthalmic and vision research.

For tumor implantation, animals were anesthetized with 100 mg/kg ketamine and 10 mg/kg xylazine and thereafter, mice were inoculated subcutaneously with 1x10^6^ Y79 cells in Matrigel (BD Biosciences, US) into the right flank. Animals were monitored for tumor growth and upon appearance of a subcutaneous mass, tumor dimensions were measured every 2 days in two perpendicular directions using digital calipers. Tumor volumes were calculated applying the formula ([LxW^2^] x 0.5), where L and W represent the largest and smallest tumor dimension, respectively. Tumors were allowed to grow until they reached about 200 mm^3^. Thereafter, animals were randomly divided into three groups: 1) Group A, control or PBS-treated animals were injected with 200 ml of saline solution; 2) Group B received a sterile solution of topotecan injected intraperitoneally (i.p) at 3 mg/kg once a week for two weeks as the maximum tolerated dose of topotecan and 3) Group C, metronomic topotecan animals were treated with an i.p daily dose of 0.6 mg/kg for 5 consecutive days a week for two consecutive weeks. Then, animals in groups B and C received the same cumulative dose of 3 mg/kg/week. The doses were selected according to the reported preclinical studies on the efficacy of topotecan against xenografts derived from several pediatric solid tumors [32]. Mice were serially weighed as a surrogate of treatment toxicity and tumor size was measured every 2 days using a caliper for efficacy assessment.

### Statistical analysis

The IC50 values were calculated by non-linear regression analysis using GraphPad Prism (GraphPad Software, La Jolla, CA, USA.). Unpaired t-test analysis was performed to compare the IC50s obtained using conventional and metronomic treatment with melphalan or topotecan in each cell model. P values of <0.05 were considered statistically significant.

One-way ANOVA was used to compare the percentage of branching among control, and topotecan or melphalan treated cells under either conventional or metronomic treatments and pair-wise comparisons between groups were performed using the Bonferroni a posterior test.

Two-way ANOVA was used to compare the tumor volume among PBS-treated, MTD and LTD tumors at different times after the start of treatment. In all cases, the statistical significance was set at p<0.05.

## Results

### Effect of treatment schedule on the sensitivity of retinoblastoma cells

**Fig 1:**
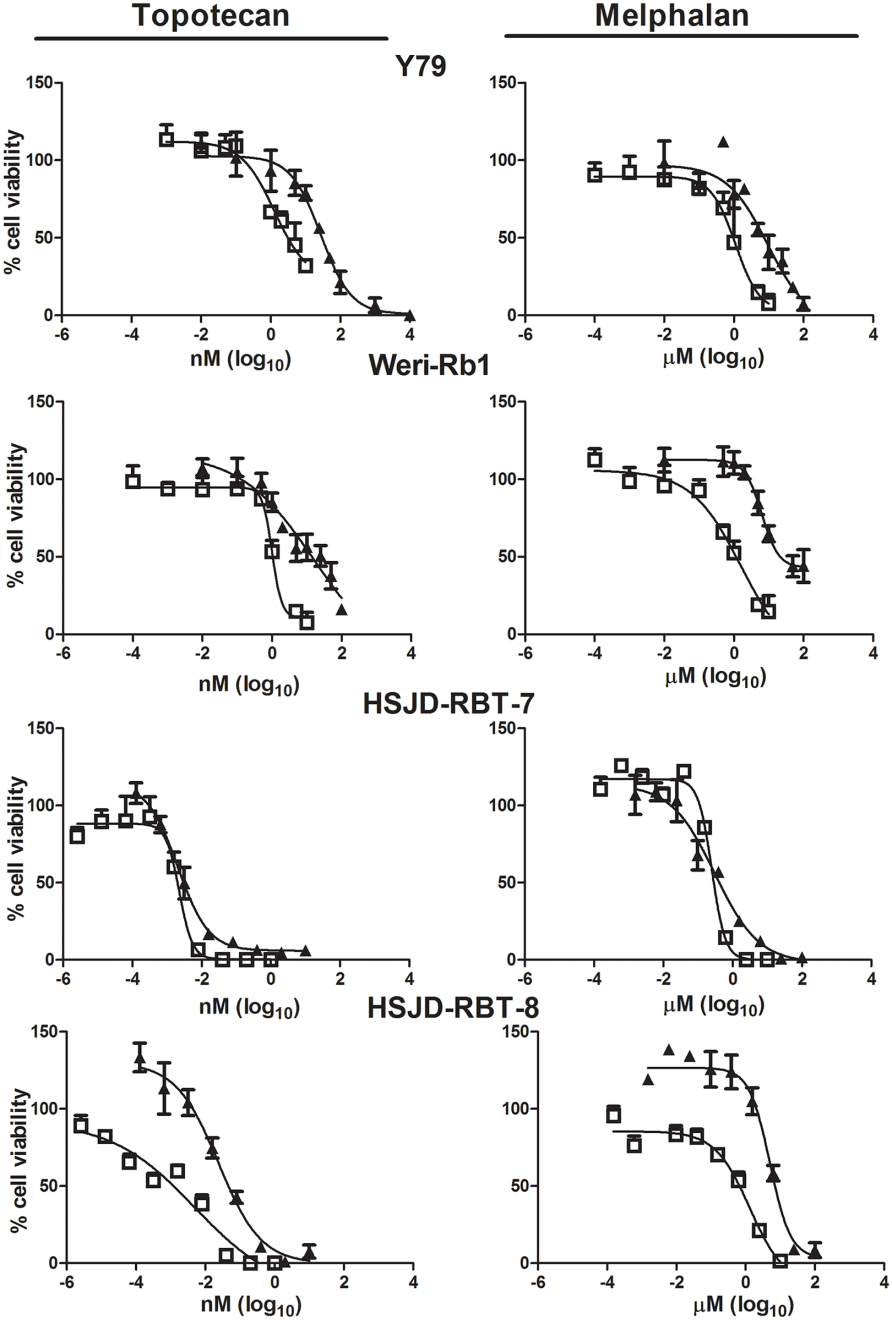
Effect of chemotherapy treatment schedule on retinoblastoma cell proliferation. Growth inhibition assay performed on Y79, WERI-RB1, HSJD-RBT-7, and HSJD-RBT-8 cell models, respectively, using MTT after 72-h incubation of a conventional regimen (full triangle symbols) and metronomic treatment (open square symbols) for 7 days with different concentrations of topotecan (left) or melphalan (right). All symbols represent % of cell proliferation as compared to untreated control cells, expressed as means (SEM) of three independent experiments, each performed in triplicate.

Commercial as well as patient-derived retinoblastoma cells showed a concentration-response cytotoxic effect to melphalan and topotecan after conventional treatment. Median IC50s after conventional treatment ranged from 3.3nM to 28.6nM and from 0.3μM to 11.9μM for topotecan and melphalan, respectively (Table 1). Of the patient-derived cell models, HSJD-RBT-7 (treatment naïve) was 10-fold and 16-fold more sensitive to topotecan and melphalan, respectively, than HSJD-RBT-8 (extensive chemotherapy treatment) (p<0.05) (Fig 1; Table 1).

**Table 1:** Cytotoxic activity of melphalan and topotecan on retinoblastoma cells after conventional and metronomic treatment.

	Topotecan			Melphalan		
Cell line	Conventional IC50 (nM)	Metronomic IC50 (nM)	IC50 Ratio	Conventional IC50 (μM)	Metronomic IC50 (μM)	IC50 Ratio
**Y79**	28.6 (26.1–31.5)	1.6^a^ (1.4–2.2)	18.4	9.7 (8.9–12.3)	1.2^a^ (0.9–2.9)	8.3
**WERI-RB1**	18.3 (16.7–20.6)	1.5^a^ (1.3–1.7)	12.3	11.9 (9.4–12.2)	0.9^a^ (0.7–1.6)	13.5
**HSJD-RBT-7**	3.3 (2.4–5.2)	2.3^c^ (1.7–2.7)	1.4	0.31 (0.21–0.62)	0.23^c^ (0.23–0.30)	1.3
**HSJD-RBT-8**	31.5^b^ (30.1–37.0)	10.3^a^^,^^b^ (6.9–11.0)	3.0	5.0^b^ (4.8–6.2)	1.3^a^ (0.5–2.0)	3.9

Data are shown as median value of 3 experiments (range).

^a^ p<0.05 compared to conventional treatment;

^b^ p<0.05 compared between patient-derived retinoblastoma cell lines after conventional topotecan or melphalan;

^c^ p>0.05 compared to conventional treatment.

Metronomic chemotherapy IC50 in Y79 and WERI-RB1 cells resulted in a significant decrease in melphalan and topotecan IC50s compared to conventional dosing with a decrease of 12- to 18-fold for topotecan and of 8.3- to 13.5-fold for melphalan as shown in Table 1 (p<0.05). Whereas a 3- to 4-fold decrease in metronomic IC50 was observed for topotecan and melphalan compared to conventional dosing in HSJD-RBT-8 (p<0.05), no change in the sensitivity to both agents was evident for HSJD-RBT-7 (p>0.05, Table 1).

### Differential sensitivity of the antiangiogenic effect of conventional and metronomic chemotherapy

**Fig 2:**
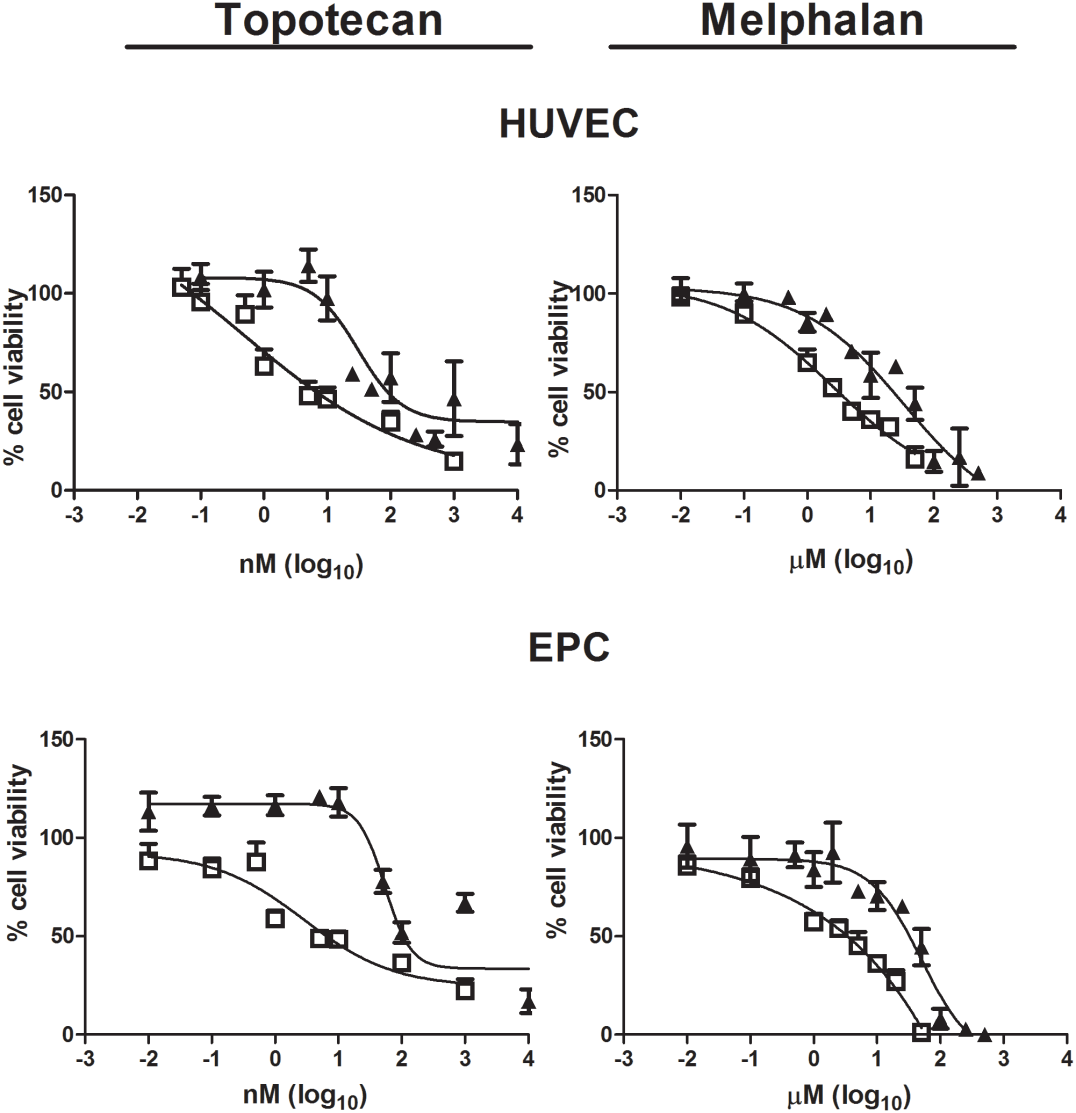
Effect of conventional and metronomic chemotherapy on proliferation of endothelial cells. Growth inhibition assay performed on HUVEC and EPC after 72-h incubation of a conventional regimen (full triangle symbols) and metronomic treatment (open square symbols) for 7 days with different concentrations of topotecan (left) or melphalan (right). Symbols represent % of cell proliferation as compared to untreated control cells, expressed as means (SEM) of three independent experiments, each performed in triplicate.

A strong and concentration-dependent antiproliferative effect was observed when EPC and HUVEC endothelial cells were exposed to conventional treatment with topotecan or melphalan (Fig 2). Similar to the antitumor effect, a differential toxic effect on endothelial cells was observed comparing metronomic to conventional treatment (Table 2) with both topotecan and melphalan. Metronomic dosing lead to lower IC50s for topotecan and melphalan (i.e. higher potency) compared to conventional exposure in both cell types (p<0.05). Metronomic treatment with topotecan decreased the IC50 23-fold and 18-fold in HUVEC and EPC cells, respectively, compared to conventional dosing. For melphalan, the IC50 decreased from 22-fold to 9-fold after continuous compared to after conventional exposure.

**Table 2:** Cytotoxic activity of melphalan and topotecan on endothelial cell types after conventional and metronomic treatment.

	Topotecan			Melphalan		
Cell line	Conventional IC50 (nM)	Metronomic IC50 (nM)	IC50 Ratio	Conventional IC50 (μM)	Metronomic IC50 (μM)	IC50 Ratio
**HUVEC**	32.5 (29.4–42.8)	1.4^a^ (1.3–2.0)	22.9	43.2 (39.8–55.3)	2.0^a^ (1.8–2.6)	21.6
**EPC**	35.8^c^ (32.9–53.2)	2.0^a^^,^^c^ (1.9–3.7)	17.6	66.7^b^ (63.9–77.5)	7.5^a^^,^ ^b^ (7.0–9.9)	8.9

Data are shown as median value of 3 experiments (range).

^a^ p<0.05 compared to conventional treatment;

^b^ p<0.05 compared between cell types under the same treatment schedule;

^c^ p>0.05 compared between cell types under the same treatment schedule.

HUVEC was the endothelial cell type most sensitive to melphalan using both conventional and metronomic treatment (p<0.05, Table 2); however, no statistical difference was observed between HUVEC and EPC topotecan IC50s after either conventional or continuous treatment (p>0.05).

Matrigel assay was performed to study the schedule-dependence of topotecan and melphalan in affecting the capacity of endothelial cells to undergo morphological differentiation into vascular structures *in vitro*. HUVEC and EPC were exposed to topotecan or melphalan at the conventional and metronomic IC50s for 72 h and 7 days, respectively, and a similar number of viable cells were then plated in matrigel. Regardless of the treatment schedule, both endothelial cell types were unable to form vascular structures. Therefore, we decided to use the maximum concentration of drugs that failed to induce cell death, corresponding to the IC15 for melphalan and the IC25 for topotecan.

**Fig 3:**
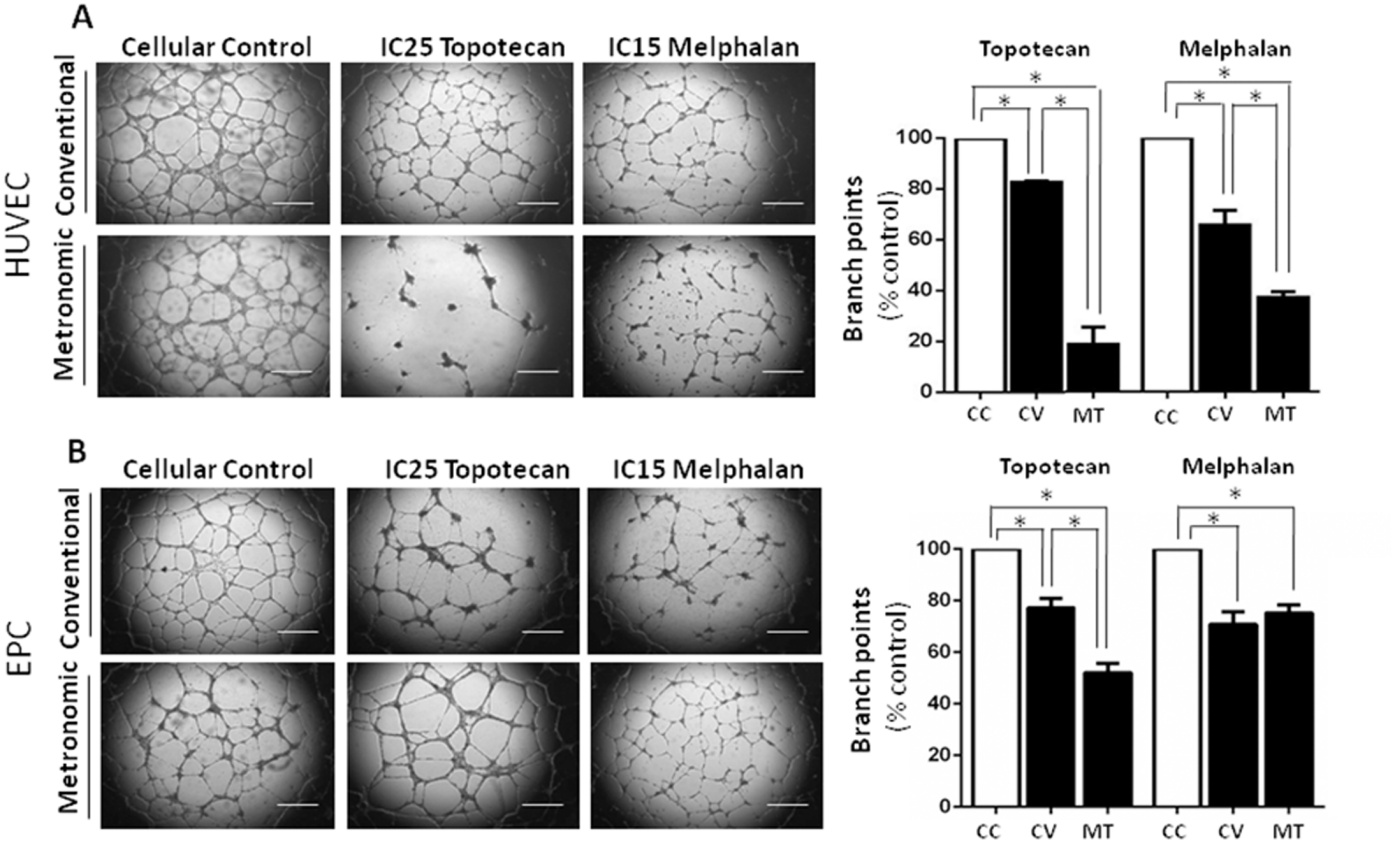
In vitro angiogenesis assay using Matrigel to characterize the antiangiogenic effect mediated by treatment scheme with topotecan and melphalan. Representative phase-contrast micrographs of tubular structures in cultured HUVEC (**A**) and EPC (**B**) previously exposed to conventional single-dose (upper row) or continuous treatment for 7 days (lower row) with topotecan at the IC25 (conventional IC25, 15nM; metronomic IC25, 0.5nM) or melphalan at the IC15 (conventional IC15, 5μM; metronomic IC15, 0.5μM), respectively. Magnification: 40X; Scale bar: 250μm. Experiments were performed in triplicate wells and five areas from each well were analyzed. The bar graph illustrates the significant decrease in the percentage of branch points after topotecan or melphalan used in a conventional or metronomic fashion compared to PBS-treated (control) cells or between treatment schedules. Data are shown as mean (SE) of three independent experiments; *p<0.05. Abbreviations: CC, control cells; CV, conventional treatment; MT, metronomic treatment.

HUVEC and EPC capillary tube formation on Matrigel was decreased by continuous treatment with topotecan to a mean (SE) percentage of branch points formation of 18.7% (6.9) and 52.0% (3.7), respectively, and statistically differed from PBS-treated cells (p<0.05). Moreover, as shown in Fig 3, tube formation in both HUVEC and EPC cultures was less after metronomic topotecan than after conventional treatment (p<0.05). Finally, the percentage of branching decreased significantly after conventional topotecan treatment of that obtained in PBS-treated cells (p<0.05).

Conventional and continuous exposure to melphalan resulted in impaired angiogenic properties in both HUVEC and EPC compared to control cells as shown for a representative experiment (Fig 3A and 3B, p<0.05). The capacity of forming capillary tubes after single-dose melphalan to HUVEC and EPC remained at 65.7% (5.8) and 70.8% (4.9), respectively, while continuous exposure to melphalan strongly inhibited tube-structure formation of these cell types reducing it to 37.0% (2.3) and 75.2% (3.2), respectively.

Although EPC showed a similar vessel number after both treatments (Fig 3B), after metronomic treatment the appearance of the vessels had deteriorated. The vessels were thinner and shorter than in controls (mean±SEM tubule length in micrometers was 34.6±6.9 and 69.2±7.7, respectively).

### Melphalan and topotecan IC50 determined by flow cytometry

**Fig 4:**
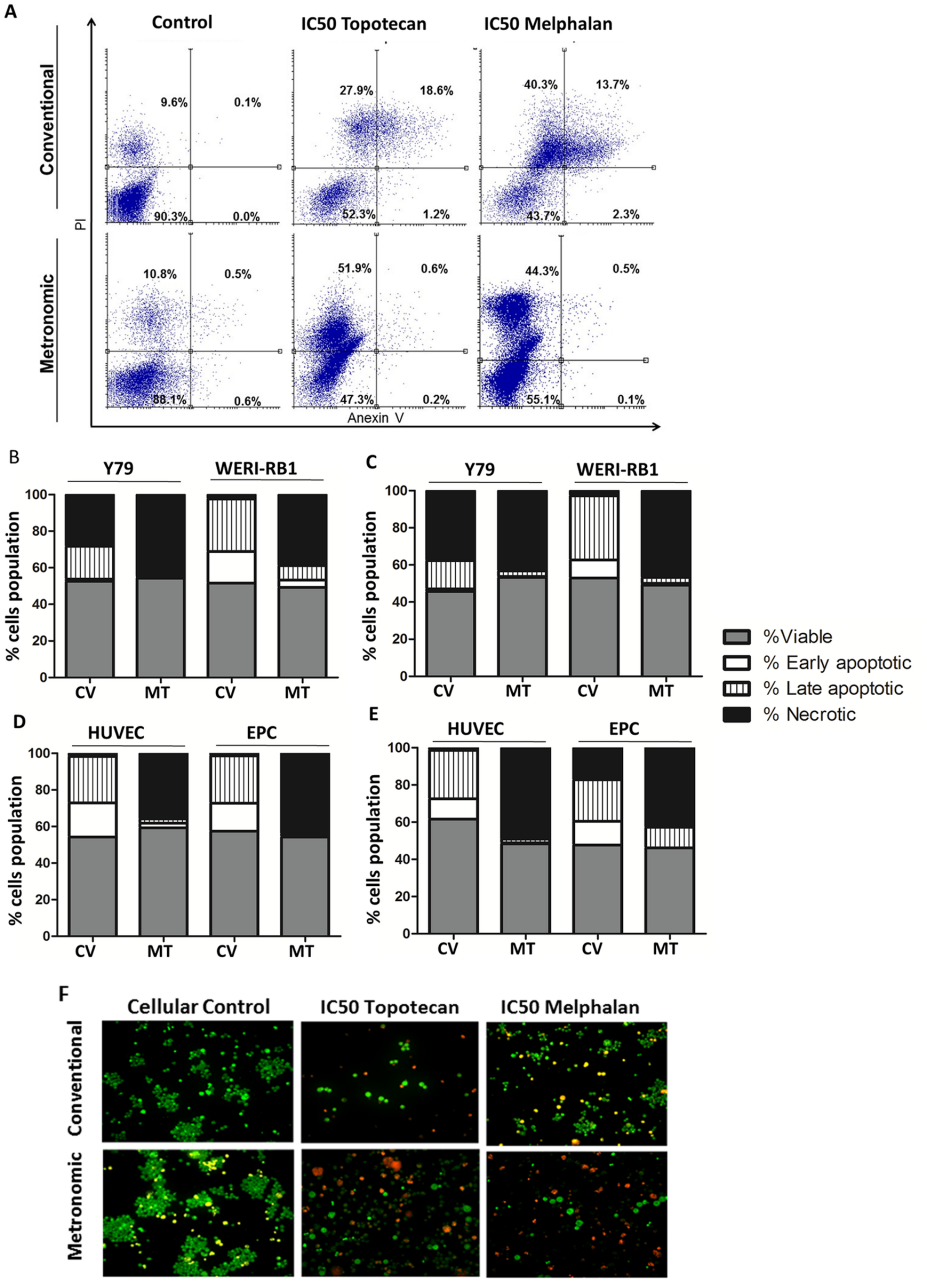
Effect of treatment schedule of chemotherapy on apoptosis of retinoblastoma and endothelial cells. Retinoblastoma and endothelial cells were cultured at the conventional or metronomic IC50 of melphalan or topotecan. The rate of apoptosis was assessed by flow cytometry after double-staining with Annexin V-FITC and PI. (**A)** Representative dot plot of Y79 after conventional and metronomic treatment with topotecan or melphalan. Lower left and right quadrants represent viable (Annexin V^-^/PI^-^) and early apoptotic (Annexin V^+^/PI^-^) cells, respectively; the upper right and left quadrants show late apoptotic (Annexin V^+^/PI^+^) and necrotic (Annexin V^-^/PI^+^) cells, respectively. Numbers in each quadrant represent percentages of cells. (**B, C**) Percentages of viable (grey bars), early apoptotic (white bars), late apoptotic (dashed bars), and necrotic cells (black bars) are shown after conventional and metronomic treatment of Y79 and WERI-RB1 with (**B**) topotecan or (**C**) melphalan. Thereafter, we studied the impact of (**D**) topotecan or (**E**) melphalan conventional and metronomic treatment on HUVEC and EPC apoptosis. (**F**) Representative images of the morphologic changes of Y79 after conventional (upper row) or continuous treatment (lower row) with melphalan or topotecan observed by fluorescent microscopy (Magnification: 200X). Each experiment was performed in triplicate and repeated three times. Percentages correspond to mean (SD). Abbreviations: CC, control cells; CV, conventional treatment; MT, metronomic schedule.

In order to confirm the IC50 previously determined by MTT assay, we tested the effects of chemotherapy treatment schedule on retinoblastoma and endothelial cells apoptosis and necrosis by flow cytometry using Annexin V and PI, markers widely used to distinguish between necrotic and apoptotic cells. Importantly, flow cytometry assays were performed at the end point of the treatments. Thus, the levels of necrosis that we observed at a single time-point could be either due to the natural outcome of an apoptotic process (which in absence of scavenger cells, proceeds to an autolytic necrotic fate called secondary necrosis) or due primary necrosis, i.e. cellular necrosis occurring *ab initio* (reviewed in Krysko D., 2008[33]). A representative graph for the results obtained with Y79 cells is shown in Fig 4A. Our data indicated that conventional and metronomic treatment with melphalan and topotecan at the previously defined IC50 for both treatment schedules induced apoptosis or necrosis in about 50% of the retinoblastoma (Fig 4B and 4C) and in both type of endothelial cell cultures (Fig 4D and 4E). Specifically, after conventional treatment of Y79 with topotecan, of the total of cells a mean of 28% cells were necrotic and only 14% were in early and late apoptosis (hereinafter referred to as apoptosis). Conversely, after metronomic treatment, of all Y79 cells 46% were in necrosis and 0.9% in apoptosis. In the case of WERI-RB1, after conventional and metronomic treatment with topotecan 39% and 11% of the cells were apoptotic while 1.5% and 38% were necrotic after each treatment, respectively. Endothelial cell lines showed a behavior that was similar to that after conventional treatment with topotecan; both cell types were mainly apoptotic (HUVEC, 44%; EPC, 41%) and changed to necrosis after metronomic topotecan (HUVEC, 36%; EPC, 48%). In addition, both conventional and metronomic melphalan in Y79 mainly led to necrosis, showing 38% and 43% of necrotic cells, respectively. However, WERI-RB1 cells showed apoptosis (41%) after conventional but necrosis following metronomic treatment (55%). Results in endothelial cells were similar, showing apoptosis of 37% and 28% of the cells after conventional dosing in HUVEC and EPC, respectively, and necrosis of 37% and 54% after the metronomic scheme. Altogether, flow cytometry confirmed the IC50 previously obtained by the proliferation assay for topotecan and melphalan in conventional or metronomic schedules. In addition, after metronomic treatment cell necrosis was mainly observed, while after conventional dosing early or late apoptosis was seen. Such differences could be the outcome either of a more effective induction of apoptosis in metronomic-treated cells compared to those under conventional treatment, or of a different mechanism of cell death induced in each case. The mechanism of cell death induced by both treatments requires further investigation which was beyond the scope of the present study.

Further confirmation of the present results was obtained by fluorescence microscopy using acridine orange/propidium iodide staining as shown for Y79 (Fig 4F). Cells exhibited chromatin condensation, shown as bright green fluorescence, and a reduction of cell size and nuclear fragmentation. Late-stage apoptosis induced by topotecan or melphalan was identified by the reddish-orange color obtained by propidium iodide binding to denatured DNA. The same morphological changes were observed in endothelial cells.

### Expression of ABC transporters after chemotherapy treatment in retinoblastoma and endothelial cell types

**Fig 5:**
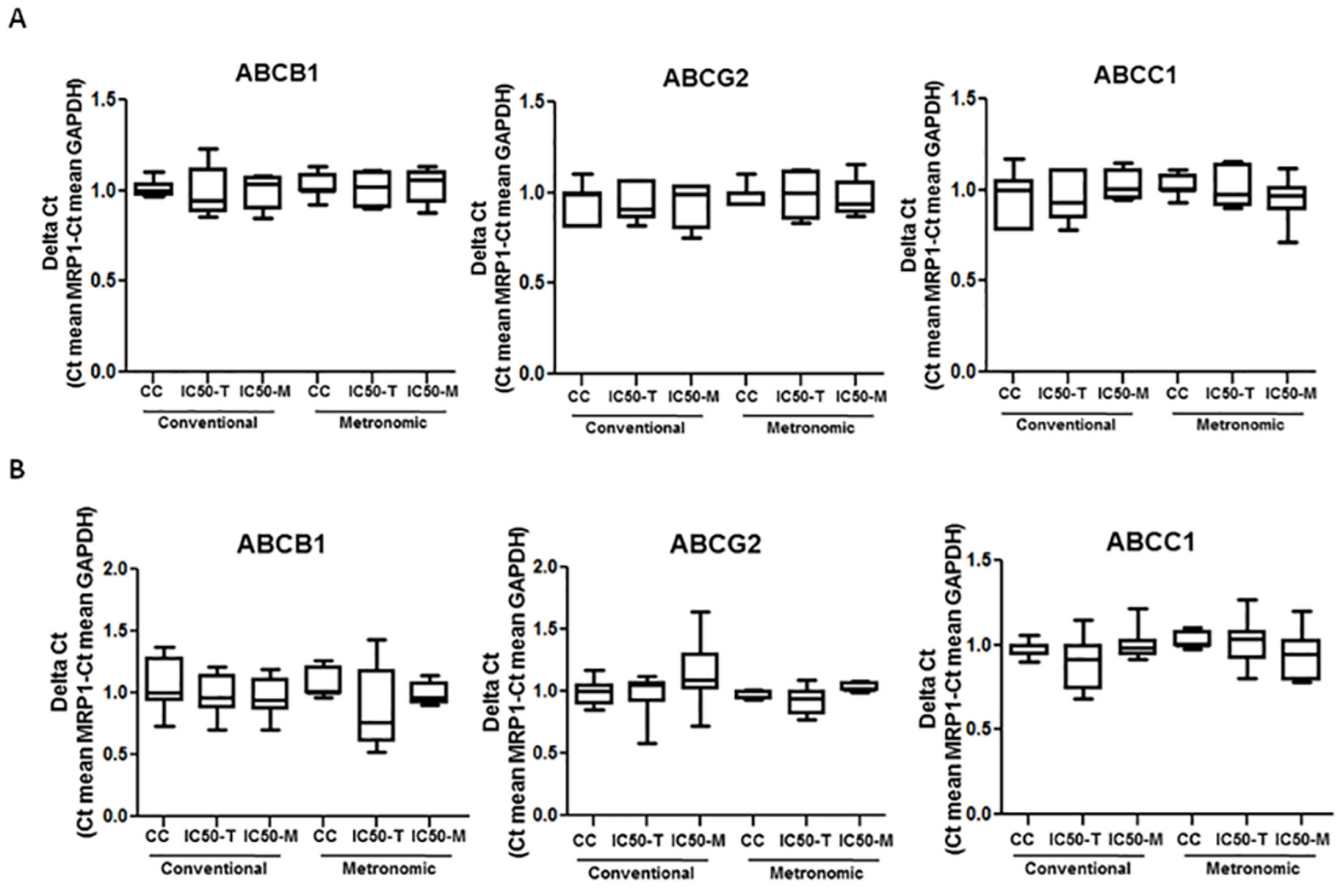
Effect of chemotherapy schedule on the expression of ABC transporters in retinoblastoma cell line Y79 and EPC cells. Relative *ABCB1* (left), *ABCC1* (middle), and *ABCG2* (right) expression level after exposing Y79 (**A**), EPC (**B**), respectively, to melphalan or topotecan conventional or metronomic treatments. All experiments were repeated at least three times. The 2−ΔΔCt method was used to analyze the relative change. Data represent mean ± SD of at least three experiments.

In order to study the effect of treatment schedule on the potential development of multidrug resistance, we characterized mRNA levels of the drug transporters ABCB1, ABCG2, and ABCC1 by means of RT-qPCR. The expression of GAPDH was used as a loading control. As shown in Fig 5A, the expression of the genes that encode the studied transporters remained unchanged as there was no change in mRNA levels in Y79 after conventional or continuous exposure to topotecan or melphalan at the IC50 calculated in the sensitivity study (p>0.05). Specifically, we observed no statistical difference when comparing the expression levels of the transporters of Y79 cells treated with LDM-IC50 topotecan or melphalan to vehicle-treated or cells exposed to MTD-IC50 (p>0.05). Moreover, the same results were found for the endothelial cell type EPC. No changes in *ABCB1*, *ABCC1* or *ABCG2* mRNA levels were found in any of the experimental conditions in which EPC were exposed to topotecan or melphalan (p>0.05, Fig 5B).

### Differential effect of metronomic and conventional treatment on xenograft tumor growth

**Fig 6:**
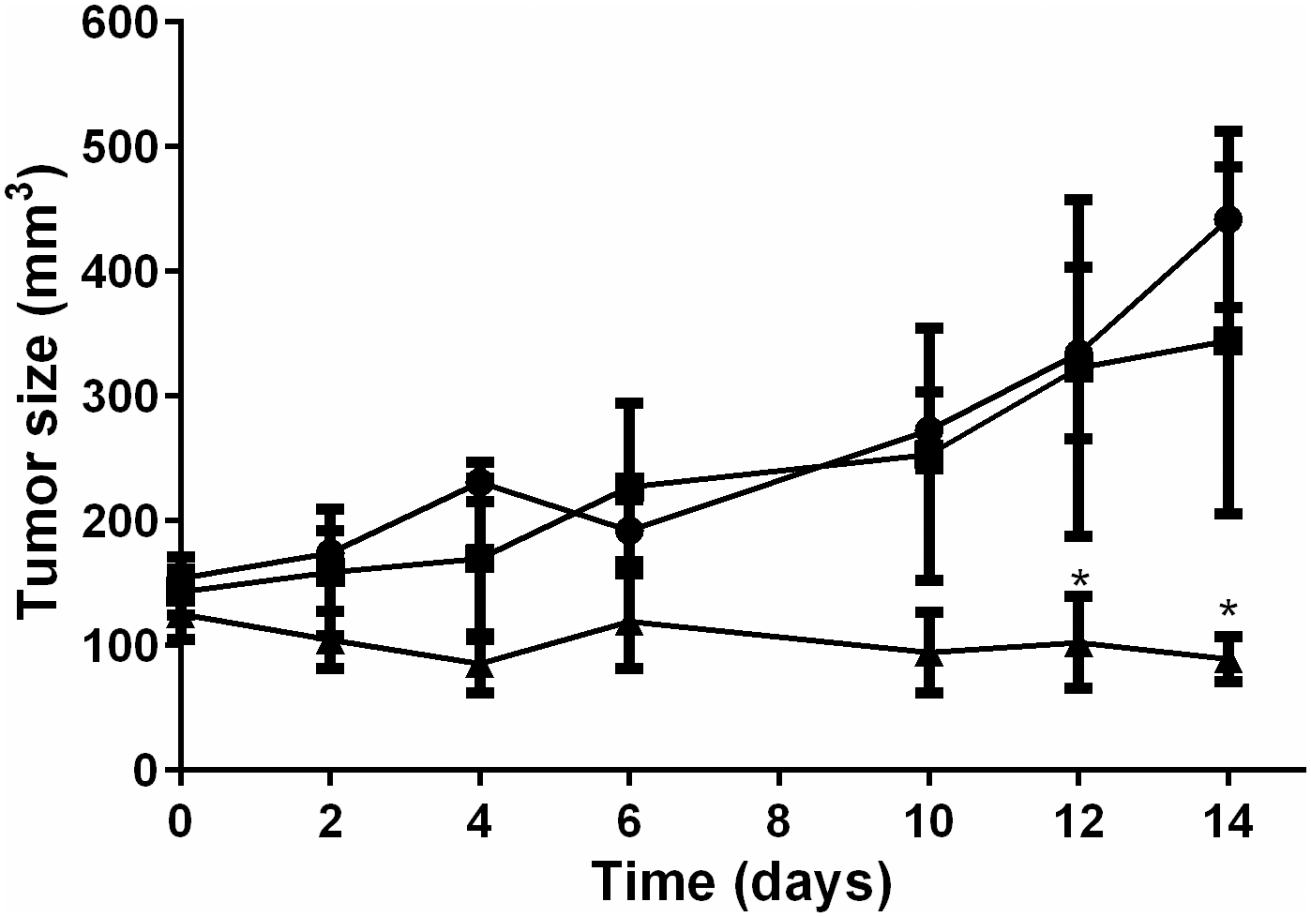
Effect of treatment on retinoblastoma xenograft tumor growth. Symbols show the tumor volume of (■) PBS-treated, (●) Maximum tolerated dose topotecan (3 mg/kg i.p once a week for two weeks), and (▲) Metronomic topotecan schedule of treatment (0.6 mg/kg i.p five days a week for two weeks). Symbols and bars represent mean ± sem. * p<0.05 with respect to maximum tolerated dose or PBS treated tumors.

Tumors in PBS-treated animals (Group A) showed progressive enlargement with a mean (SD) tumor volume of 442.0mm^3^ (141.5) 14 days after the start of the injections. As shown in Fig 6, after two weeks of chemotherapy treatment mean (SD) tumor volume of retinoblastoma xenografts in MTD (group B) and metronomic (group C) topotecan treated animals was 344.7 mm^3^ (278.2) and 89.2 mm^3^ (36.4), respectively. Thus, a significant difference was observed in tumor volume 12 days after the start of topotecan injections which remained significant thereafter comparing between groups B and C and between groups A and C (p<0.05). No significant difference in tumor volumes was detected between group A and B animals at all times after the start of treatment (p>0.05).

The toxicity profiles were similar among the three studied groups as no weight loss and significant difference in animal weights were observed during treatment among the three study groups (p>0.05). Mean (SD) animal weight before the first injection was 23.4g (3.1), 29.1g (1.4) and 21.9g (1.1) and at completion of the study it was 23.7g (3.9), 22.3g (2.3), and 22.3g (2.4) for Group A, B and C animals, respectively.

## Discussion

Current pharmacological treatment of retinoblastoma relies on high doses of chemotherapy. In some pediatric malignancies, continuous administration of low doses, also referred to as metronomic chemotherapy, provides an alternative to maximum tolerated doses showing better tumor control and a lower rate of adverse events. For retinoblastoma treatment, this information may be useful for treating slowly dividing cells from vitreous seeds or as maintenance therapy after complete remission was achieved with intensive chemotherapy. Hence, our aim was to study the cytotoxic effects of continuous exposure to topotecan or melphalan, routinely used in the clinics, in retinoblastoma cells and the antiangiogenic effect of this modality in comparison to single-dose exposure. Our results suggest that continuous chemotherapy treatment increases the sensitivity to the cytotoxic and antiangiogenic effect of both agents compared to single-dose exposure without inducing multi-drug resistance.

The administration of the maximum tolerated dose is the most commonly used modality in clinical practice in oncology disregarding the dose intensity and schedule[34,35]. However, this approach may lead to ocular toxicity after intravitreal injections of chemotherapy or even life-threatening systemic toxicities after ophthalmic artery chemosurgery or intravenous infusion[3,9–12]. These adverse events could be avoided if lowering the dose but prolonging the time of administration of the pharmacologically active drug also referred to as metronomic treatment[14,15,36]. Evidence suggests that metronomic chemotherapy is promising in pediatric oncology in terms of efficacy and safety and thus, it would be interesting to assess its value in intraocular retinoblastoma treatment[14,17,37]. An alternative to repetitive intravitreal administrations could be a sustained-release formulation delivered periocularly or inserted in the vitreous cavity as previously attempted[25,38,39]. Nonetheless, further design and development of such a device would be necessary and this topic is beyond the aim of the present study. Moreover, patients with disseminated disease mostly live in developing countries and may largely benefit from metronomic chemotherapy based on its low-cost and easy access treatment as previously reported for agents other than topotecan and melphalan in this clinical setting[3,19,20,40].

The potent preclinical activity of topotecan as a single-agent in retinoblastoma has not been completely replicated in the clinical setting after local administration[5,41]. Thus, its use is commonly restricted to concomitant administration of melphalan or carboplatin[42–45]. Such limited activity may be related to the inappropriate schedule of administration in retinoblastoma as a once-weekly maximum dose, based on its cell-cycle dependent mechanism of action mediated by impairment of topoisomerase-I activity and DNA synthesis inhibition. As opposed to single-dose maximum exposure, low daily topotecan doses *in vitro* or dosages in xenografts derived from pediatric solid tumors showed a strong antitumor response supporting the hypothesis that topotecan activity is highly schedule- and dose-dependent[32,46]. Subsequently, a protracted schedule of topotecan was evaluated in children with high-risk neuroblastoma with improved clinical response[47]. Additionally, pharmacokinetic/pharmacodynamic simulations showed the benefits of protracted topotecan schedules in terms of antitumor activity and decreased incidence of myelosuppression in neuroblastoma[48]. Accordingly, our data also showed a 12 to 18-fold reduction in the IC50 upon exposure of cells to continuous topotecan for 7 days as compared to single-dose exposure. Also, we validated the *in vitro* observations with *in vivo* results obtained in a retinoblastoma xenograft model. Low-dose continuous topotecan exposure (5 days for 2 weeks) allowed controlling tumor growth showing a significant difference in tumor volumes compared to the animals exposed to the same total topotecan dose but given in a maximum-tolerated-dose schedule. Therefore, our hypothesis on a differential tumor growth control depending on the schedule of topotecan administration was further confirmed for the first time in an animal model of retinoblastoma supporting the higher efficacy of low and continuous dosing in this tumor. Furthermore, in a recent study on the role of metronomic topotecan in preclinical models of neuroblastoma, the authors showed that metronomic topotecan differentially promoted a tumor-inhibiting type of senescence as a favorable mechanism of cell death *in vitro* and prolonged survival in *in vivo* models [21]. Whether this is the case for retinoblastoma is an important issue that requires further investigation.

The use of low-passages patient-derived retinoblastoma cell models strengthens our study as they were derived from both naive and heavily-pretreated patients. Moreover, these cell models were established in serum-free medium which promotes the growth of tumorspheres that conserve the original genetic properties of the tumor of the patient[49]. In contrast, established cell lines grown in serum medium even for decades, are more prone to contain variations of the original tumor properties[50]. In this sense, a distinct phenotype related to invasiveness has been shown for commercial retinoblastoma cell lines compared to patient-derived cell models though extensively used for pharmacological assessment[51,52,53]. Thus, it is conceivable that changes in drug administration schedules distinctively impact the IC50, a parameter of chemosensitivity phenotype, as we observed a more pronounced effect of metronomic treatment in cell lines compared to the HSJD-RBT-8 cell model (Table 1). In addition, single-dose topotecan was around 10-fold more active in HSJD-RBT-7 cells (naive) than in HSJD-RBT-8 (heavily pretreated), suggesting that selection of resistant clones by currently used clinical treatments based on conventional schedules of chemotherapy might be involved in the development of chemoresistance and should be further assessed. We also highlight the finding that HSJD-RBT-008 cells showed increased chemosensitivity to the metronomic schedule with a lower IC50 of both drugs compared to conventional treatment[53]. Thus, a potential way to overcome resistance in the clinics would be using continuous treatment with melphalan or topotecan through a direct cytotoxic effect against retinoblastoma cells.

Melphalan, as cyclophosphamide, belongs to the nitrogen mustard alkylating agents and is widely used for intraocular retinoblastoma treatment by local delivery routes[4,54,55]. The dose and schedule for melphalan delivery is currently based on the clinical experience of the treating team considering a balance between tumor control and systemic or ocular toxicity[3]. Despite excellent antitumor response, ocular anterior and posterior chamber toxicities and systemic neutropenia have been documented and thus, a deeper understanding and definition of the range for melphalan dose would improve patient outcome[9–11]. As metronomic use of another alkylating agent, cyclophosphamide, improves antitumor response in different solid tumors, we reasoned that a similar schedule could enhance the activity of melphalan in retinoblastoma[56]. In our study we observed a significant reduction in melphalan IC50 after continuous exposure compared to single-dose treatment in all cell models but HSJD-RBT-7. Taken together, our results suggest that tumor control in patients who relapsed after having previously been treated with these agents in a conventional maximum-dose fashion may be attained using metronomic treatment. Another interesting finding was the significant difference in the sensitivity to melphalan found between both patient-derived cell lines possibly resulting from changes in the intracellular pathways. This difference in sensitivity should be further studied.

There is a growing body of literature on the advantages of combining anticancer agents with antiangiogenic drugs[57–59]. However, if the chemotherapy agent also displays antiangiogenic activity at a relatively similar concentration, it would be an additional benefit for patient treatment simplicity. Based on the well-documented role of tumor angiogenesis in retinoblastoma we studied the effect of chemotherapy exposure schedule in two endothelial cell types. In a previous toxicity study in rabbits melphalan exerted a not well-defined toxic effect on the retinal and choroidal vascular endothelial cells[10]. Furthermore, it has been recently reported that melphalan may trigger endothelial vascular toxicity by deregulation of MYC and NF-kb1 transcription factor intracellular pathways[60]. In our study we show for the first time that melphalan exerts a potent antiangiogenic effect in mature (HUVEC) and endothelial progenitor cells (EPC). The present results show that metronomic treatment highly improved antiangiogenic activity compared to single-dose exposure with a 9- to 22-fold increase in the IC50. In addition, continuous exposure to melphalan impaired the capacity of endothelial cells to form vascular structures on Matrigel supporting the use of metronomic melphalan. Therefore, our results for melphalan are in agreement with those previously reported on the antiangiogenic effect of metronomic cyclophosphamide *in vitro* and *in vivo* [55]. Moreover, our findings agree with previous studies that showed the schedule-dependent impairment of angiogenesis to long-term vinblastine exposure favoring the use of metronomic chemotherapy[61].

Consistent with previous studies we observed an antiangiogenic effect of topotecan in both single and continuous dosing *in vitro*. The antiangiogenic effect of topotecan in HUVEC cells (between 12nM and 50 nM) and in an animal model of angiogenesis has been reported[62,63]. In our study, in endothelial cell types, the topotecan IC50 was about 30nM after single-dose exposure, closely resembling results of previous reports. In addition, a strikingly significant increase in vascular endothelial cell sensitivity to topotecan was observed after continuous exposure with an 18- to 23-fold decrease in the IC50 compared to a 10-fold decrease reported by others[63]. This discrepancy may result from experimental conditions including the time interval in which the culture was exposed to both single and continuous topotecan dosing. Finally, it is interesting to note that topotecan continuous exposure exerted an antiangiogenic effect at an IC50 comparable to that attained in commercial retinoblastoma cells. Thus, if translated into *in vivo* treatment, continuous topotecan would have a double antiangiogenic and direct antitumor effect. Moreover, both HUVEC and EPC capillary-like tube formation was severely impaired after continuous exposure to the agent supporting the hypothesis of the schedule-dependent antiangiogenic activity of topotecan that may be overlooked as lack of activity if administered using a conventional schedule.

Both topotecan and melphalan are substrates of the ABCB1 gene product, P-glycoprotein. In addition, topotecan is a well-documented substrate for ABCG2 and ABCC1 transporters playing important roles in topotecan disposition in animal models[29,64–67]. Specifically, enucleated eyes from chemotherapy-naive retinoblastoma patients showed high ABCB1 expression and thus, the resistance mechanisms that involve ATP transporters may play an important role in the disposition of chemotherapy in the context of ocular tumor control[67]. Still, it is controversial the effect of the schedule of chemotherapy exposure in the expression of ABC transporters in retinoblastoma patients while a major impact is well documented in ovarian cancer cell lines and vascular endothelial cells[68,69]. Specifically, Pasquier *et al* reported a significant increase in *ABCB1* but not in *ABCC2* expression in endothelial cells irrespective to the long-term treatment schedule at the MTD or LTM with vinblastine or etoposide[61]. Therefore, our study sought to determine the effect of single *versus* continuous exposure to melphalan or topotecan on the expression of ABC transporters in retinoblastoma and endothelial cells as a surrogate of chemotherapy resistance and dependence of drug treatment schedule.

Our results showed that single exposure to chemotherapy or even longer and continuous treatments with melphalan or topotecan at the IC50 did not alter *ABCB1*, *ABCC1*, or *ABCG2* expression levels in retinoblastoma and HUVEC cells. Phenotypes of drug resistance have been previously developed by exposing tumor and endothelial cells or tumor-bearing animal models to different schedules of chemotherapy[27,69–71]. Nonetheless, our study involved continuous melphalan or topotecan exposure for 7 days. Moreover, endothelial cells may acquire resistance after exposure to different chemotherapeutic agents. Nonetheless, we did not observe any change in ABC transporters expression levels in HUVEC and EPC after continuous or single dose administration or comparing treatment schedules. Therefore, exposure of endothelial cells to topotecan or melphalan for 7 days did not contribute to cell resistance due to ABC transporters over-expression in the present *in vitro* models.

In conclusion, our results showed increased melphalan and topotecan sensitivity after continuous exposure compared to single-dose exposure in both retinoblastoma and endothelial cell types without inducing multi-drug resistance protein expression. Both agents led to retinoblastoma and endothelial cell death through apoptosis and necrosis after continuous or single-dose treatment but a more pronounced cytotoxic effect with higher potency was attained after continuous treatment. We also highlight the potent cytotoxic and antiangiogenic effect of metronomic topotecan compared to single-dose for better tumor control. Finally, both melphalan and topotecan were active in two patient-derived retinoblastoma cell models showing differences between cell lines and schemes that should be further explored.
